# Formal comment on: Myhrvold (2016) Dinosaur metabolism and the allometry of maximum growth rate. *PLoS ONE*; 11(11): e0163205

**DOI:** 10.1371/journal.pone.0184756

**Published:** 2018-02-28

**Authors:** Eva Maria Griebeler, Jan Werner

**Affiliations:** Institute of Organismic and Molecular Evolution, Evolutionary Ecology, Johannes Gutenberg-University of Mainz, Mainz, Germany; Perot Museum of Nature and Science, UNITED STATES

## Abstract

In his 2016 paper, Myhrvold criticized ours from 2014 on maximum growth rates (G_max_, maximum gain in body mass observed within a time unit throughout an individual’s ontogeny) and thermoregulation strategies (ectothermy, endothermy) of 17 dinosaurs. In our paper, we showed that G_max_ values of similar-sized extant ectothermic and endothermic vertebrates overlap. This strongly questions a correct assignment of a thermoregulation strategy to a dinosaur only based on its G_max_ and (adult) body mass (M). Contrary, G_max_ separated similar-sized extant reptiles and birds (Sauropsida) and G_max_ values of our studied dinosaurs were similar to those seen in extant similar-sized (if necessary scaled-up) fast growing ectothermic reptiles. Myhrvold examined two hypotheses (H1 and H2) regarding our study. However, we did neither infer dinosaurian thermoregulation strategies from group-wide averages (H1) nor were our results based on that G_max_ and metabolic rate (MR) are related (H2). In order to assess whether single dinosaurian G_max_ values fit to those of extant endotherms (birds) or of ectotherms (reptiles), we already used a method suggested by Myhrvold to avoid H1, and we only discussed pros and cons of a relation between G_max_ and MR and did not apply it (H2). We appreciate Myhrvold’s efforts in eliminating the correlation between G_max_ and M in order to statistically improve vertebrate scaling regressions on maximum gain in body mass. However, we show here that his mass-specific maximum growth rate (kC) replacing G_max_ (= MkC) does not model the expected higher mass gain in larger than in smaller species for any set of species. We also comment on, why we considered extant reptiles and birds as reference models for extinct dinosaurs and why we used phylogenetically-informed regression analysis throughout our study. Finally, we question several arguments given in Myhrvold in order to support his results.

## Introduction

The thermal physiology of extinct dinosaurs is highly discussed and the debate mainly focuses on whether some dinosaurian taxa had been endothermic [1–10]. Scaling regressions on maximum ontogenetic growth rate and (adult) body mass of extant ectothermic and endothermic vertebrates are often used to assess the thermal physiology of extinct dinosaurs [7,8,11–16]. However, in extant species, the process of thermoregulation is very complex and reflects strikingly different metabolic situations [3,17] and also growth strategies differ between ectothermic and endothermic extant vertebrates [18].

In the first section of this introduction, we will summarize information on thermal physiology and growth in extant vertebrates. We think this background is important to understand why a simple endotherm/ectotherm dichotomy is too simplistic in terms of body temperature and metabolic rate, and why a link between the maximum growth rate and the thermoregulation strategy employed by an animal is unclear. In the next section, we will summarize the content of our study with respect to the critiques raised in Myhrvold [10], and will end with a section summarizing the issues in his paper relevant for our formal comment.

### Thermal physiology and growth in vertebrates

In extant ectotherms body temperature fluctuates and the main source of internal heat comes from the environment. Animals can thermoregulate behaviourally by moving between different microhabitats [19,20]. Basking in the sun or cooling in the water is the most typical thermal behaviour seen in reptiles [21]. It allows ectothermic reptiles to have body temperatures even more than 20°C higher than ambient air temperature, and similar to those seen in endothermic birds and mammals [22,23]. Both endothermic and ectothermic animals can bridge adverse conditions by being inactive and ceasing feeding (diurnal or seasonal torpor, hibernation throughout winter, estivation throughout summer) or by migrating to more favourable habitats [19,20,24]. In both ectotherms and endotherms internal heat sources exist that affect their body temperatures as heat is produced by all metabolic activities of an organism, but only in endothermic birds and mammals the thoracic and abdominal organs (e.g. gut, liver, kidney, heart, and lungs) produce a substantial larger amount of metabolic heat than in ectotherms [20]. The latter is reflected in that these organs have substantially higher mitochondrial densities and activities of mitochondrial enzymes in endotherms than in similar-sized ectotherms [25]. Thus, contrary to ectothermic animals, extant endothermic mammals and birds show rather constant core temperatures that they generate metabolically and that they strongly defend over a broad range of ambient temperatures against the environment [19].

In ectotherms, body temperature and metabolic rate are tightly linked and both are mainly set by the temperature to which the animal is currently exposed [19]. Contrary, in endotherms, only when ambient temperatures are close to the animal’s preferred body temperature (that in the thermoneutral zone, no regulatory changes in metabolic heat production or evaporative heat loss are needed) its metabolic rate relates to its core temperature. The metabolic rate of endotherms considerably increases compared to that of an ectothermic animal of similar size and of an identical body temperature when ambient temperatures are lower or higher than their preferred body temperature in order to keep their core temperature [26–28]. Also being endothermic comes at an extra metabolic cost (leakiness of mitochondria membranes, [29–31]). This cost is reflected in a higher metabolic rate seen in an endothermic animal (that in the thermoneutral zone) than in an ectothermic (at rest) of similar size and body temperature.

Endothermy is not the only mechanism in animals leading to quite constant and high body temperatures. Ectothermic animals living under rather constant ambient temperatures (e.g. in the deep sea) have also rather constant body temperatures. Tuna and lamnid sharks can maintain the temperature of their muscles considerable above the temperature of the ambient seawater by conserving the heat generated by normal metabolism (rete mirabile, principle of countervailing influence). Contrary to endotherms, tuna and lamnid sharks are only able to heat up certain parts of their body and they can do this only under a relatively narrow range of ambient temperatures [19,20]. The “inertial homeothermy hypothesis” works only for larger ectothermic animals (gigantothermy, [1,2]). Larger ectotherms heat up and cool down slower than smaller ectotherms, since the surface-to-volume ratio of animals decreases with increasing body mass. In other words, in ectothermic animals body temperatures increase and body temperature fluctuations decrease with increasing body mass [3]. Thus, although being ectothermic a large dinosaur (e.g. a sauropod) could have had a high and rather stable body temperature only due to its large size. Ectothermic animals exploiting any of all these options enabling rather constant body temperatures or having at least in some parts of their body higher temperatures than the environment, contrary to endotherms do not (costly) metabolically defend their body temperature against environmental temperatures [19].

The growth curve established for a single individual describes at a macroscopic level how it had increased its body mass between birth and death. In both ectothermic and endothermic vertebrates intraspecific variability in individual growth curves exists at the species and even at the population level. A few examples may illustrate how intraspecific but also interspecific variability in growth curves of animals can arise. The energy/tissue that an animal is able to allocate to growth at an arbitrary ontogenetic age depends not only on its genetic constitution, but also on the environmental conditions to which it is currently exposed and its internal status [18]. Many animals switch their diet over ontogeny (e.g. some mammals from lactation to herbivory, some birds from insectivory to gramnivory) and their subsequent growth rates are altered due to differences in the energy-content of food [32–34]. Environmental conditions, which vary over time (e.g. diurnal, seasonal, or annually), set the level of food availability and thus how well young and adults are nourished [35–37] and in turn how much energy they can allocate to growth. For example, the most northerly distributed extant crocodilian species, the American Alligator, stops eating when ambient temperature drops below 16°C. It is only during the warmer months of the year during active feeding that this species grows [38]. In the golden-mantled ground squirrel (*Spermophilus lateralis*) somatic growth is considerably reduced during hibernation due to the high risk of starvation in winter and this growth reduction coincides with a not detectable concentration of the insulin-like growth factor (IGF) [39]. Contrary to endothermic vertebrates, ectothermic vertebrates are indeterminate growers, they can grow during their complete life. Thus, ontogenetic growth in ectothermic vertebrates is much stronger shaped by environmental conditions than in endothermic animals (determinate growers) attaining a more or less fixed adult size at a comparative earlier age in their life than ectotherms [17,32]. Additionally, in most mammals and birds (determinate grower) all growth occurs before reproduction starts, while in fish and reptiles (indeterminate growers), individuals continue to grow after their first reproduction. Thus, in adult fish and reptiles, contrary to adult mammals and birds, energy/tissue is not only allocated to growth and maintenance, but also to reproduction. Altricial birds [40] and mammals [41–43] achieve full endothermy not before they are fully-grown. This allows altricial birds (mammals) to save energy compared to similar-sized precocial birds (mammals), which in turn they use to increase their growth rates [40,43].

Different studies tried to assess dinosaurian thermoregulation strategies and metabolic rates from maximum growth rates (i.e. the maximum gain in body mass per time unit observed during an individual’s ontogenetic growth, e.g. kg per year) by comparing their growth rates to those seen in similar-sized species (if necessary scaled-up) from different extant vertebrate taxa [7,8,11–16]. These studies trace back to two papers that Case published in 1978. In the first [44], he presented scaling relationships on maximum growth rate and adult body mass for different endothermic and ectothermic vertebrate groups (extant species), and in the second [45] he speculated on a link between this rate and endothermy. Consistent with the different metabolic situations and differences in growth strategies observed in extant endothermic and ectothermic vertebrates, and after having discussed pros and cons of a potential link between metabolic rate and growth rate, he concluded in his second paper *“that an organism’s growth rate is not solely determined by its metabolic rate*, *although the evolutionary achievement of endothermy seems to have resulted in lifting the physiological restraints upon growth rate enough to produce nearly a ten-fold increase over ectothermic growth rates”* [45]. Subsequent empirical studies on maximum growth rates of dinosaurs either showed plots with the regression lines on different extant vertebrate groups published by Case [44] and with maximum growth rates of dinosaurs plotted therein (e.g. [7,8]), or they straightforward used his regressions to derive the thermoregulation strategy and metabolic rate (MR) of dinosaurs from their size and maximum growth rates (e.g. [14,46]). All these studies tacitly assumed that differences in maximum growth rates of extant ectotherms and endotherms are large enough to justify the omission of factors influencing their maximum growth rates (see above) and potentially their MR (see above), except for their (adult or asymptotic) body mass (M). However, whether maximum growth rate, thermoregulation strategies and MR are indeed linked as assumed by all these studies was never tested at that time [7,8,10].

### The study of Werner and Griebeler [8]

The study of Werner and Griebeler [8] and that of Grady et al. [7] were the first aiming directly at maximum growth rate and thermoregulation strategies in vertebrates. Our study was possible because since Case’s papers [44,45] bone histology had allowed to estimate maximum growth rates of several dinosaurs (e.g. [11–14,16]), information on extant vertebrate maximum growth rates had increased in-between (especially Stark and Ricklefs [47] for birds, Zullinger [48] for mammals, and Pauly [49] for fish), and more sophisticated methods correcting regressions for the shared evolutionary history of species had been developed (e.g. [50–52]). Our study pursued three aims. First, we established new phylogenetically-informed regression lines on fish, reptiles, marsupials, eutherians, precocial and altricial birds in order to check or improve those given in Case [44] (first aim). With our new dataset we then assessed whether “*growth rates of recent taxa are unequivocally separated between endotherms and ectotherms*” [8] or in other words whether maximum growth rates of similar-sized ectothermic and endothermic vertebrate species show no overlap (second aim)? An overlap in ectothermic and endothermic maximum growth rates directly rejects the speculation given in Case [45] (he already observed some overlap in growth rates of ectothermic and endothermic species), irrespective of whether these vertebrate groups are indeed suitable reference groups for dinosaurs (see section “Groups used as references for dinosaurs”). It also removes any basis for subsequent paleontological studies on dinosaurian thermoregulation strategies making use of to this unproven speculation (e.g. [11–15]). We showed in our paper that “*on average*, *maximum growth rates of ectotherms were about 10 (reptiles) to 20 (fishes) times (in comparison to mammals) or even 45 (reptiles) to 100 (fishes) times (in comparison to birds) lower than in endotherms*” [8] and thus corroborated Case [44,45] at the level of averages in maximum growth rates of similar-sized species from different extant vertebrate groups (“*the evolutionary achievement of endothermy seems to have resulted in lifting the physiological restraints upon growth rate enough to produce nearly a ten-fold increase over ectothermic growth rates”* [45]). However, we also showed that “*individual growth rates overlapped between several taxa and even between endotherms and ectotherms*” [8]. Our results, thus clearly indicated that we cannot infer the thermoregulation strategy of any extant animal from its maximum growth rate by comparing its rate to that of any extant similar-sized (if necessary scaled-up) endotherm (mammal or bird) and to any similar-sized ectotherm (fish or reptile).

The third aim of our study was to assess whether maximum growth rates of similar-sized extant reptiles and birds overlap, too (if ectotherms and endotherms would have shown no overlap our third aim would have been redundant). We think that distinguishing only between endothermic and ectothermic vertebrates (as done in Myhrvold [10]) is unjustified from a phylogenetic and physiological perspective (see section “Groups used as references for dinosaurs”). As our analysis showed that maximum growth rates of studied ectothermic reptile and of endothermic bird species (Sauropsida model) did not overlap (no overlap in species’ maximum growth rates between reptiles and precocial/altricial birds; regression lines of reptiles and of precocial/altricial birds statistically differed, please note that slopes were statistically not different and only intercepts statistically differed between vertebrate groups), we went further on and individually checked for each of our 17 dinosaurian specimens under study whether its rate fitted to the reptile or bird model or even to none of both. As body mass ranges considerably differed between extinct dinosaurs, extant reptiles and extant birds, and we aimed to avoid a statistical incorrect extrapolation of extant groups to larger extinct dinosaurs, we used the highest positive and highest negative residual value calculated for reptiles and also for birds in order to capture the total variability seen around the average (regression line) in maximum growth rates within each of both extant groups. Our approach is in fact the method suggested by Myhrvold [10] to avoid ecological fallacy (H1, see section “Ecological fallacy H1”) and that he described as “*a parallelogram bounded above by the respective regression line shifted vertically to capture the highest positive residual and bounded below by the regression line shifted to capture the smallest negative residual*”. From the maximum growth rates of the 17 dinosaurs, the variability seen in this rate in extant vertebrates, and “*under the assumption that growth rate and metabolic rate are indeed linked*” (we were aware of that this is controversial and provided many arguments against this link, they are found in pages 6 and 7 of Werner and Griebeler [8], see also section “Ecological fallacy H2 and the Metabolic Theory of Ecology (MTE, [53])”, [54]), we concluded from our analysis: “*Compared to other sauropsids*, *the growth rates of studied dinosaurs clearly indicate that they had an ectothermic rather than an endothermic metabolic rate* (maximum growth rate). *Compared to other vertebrate growth rates*, *the overall high variability in growth rates of extant groups and the high overlap between individual growth rates of endothermic and ectothermic extant species make it impossible to rule out either of the two thermoregulation strategies for* (any of) *the studied dinosaurs*. [8]”.

Our initial analyses on the accuracy of Case’s regressions [44] (first aim, larger dataset on vertebrates, phylogenetically-informed regression analysis) uncovered an important problem with respect to high growth rates and potential endothermy in extinct dinosaurs. For extant precocial birds we found a strikingly different regression line. The regression line in Case [44] had a much lower slope than ours, whereas intercepts were comparatively similar. Thus, for small precocial birds, maximum growth rates are overestimated and for large ones they are underestimated by Case’s [44] equation (see third figure in Werner and Griebeler [8]). Consequently, under Case’s [44] maximum growth rate regression on precocial birds, rates of many large dinosaurs are consistent with those expected for (if required scaled-up) endothermic precocial birds (e.g. [11,14,15]). We anticipate that this could have added to the popularity of the hypothesis that some extinct dinosaur taxa were endothermic and had growth and metabolic rates as seen in endothermic precocial birds. The revised scaling regressions on vertebrate groups that we established in our paper [8] suggested that our studied dinosaurs (they covered several specimens for which Case’s original regression line pointed to endothermy, [44]) had maximum growth rates intermediary to those seen in ectothermic and endothermic vertebrates. Under our Sauropsida model their rates conformed best to those of extant similar-sized (if necessary scaled-up) fast growing ectothermic reptiles.

## The study of Myhrvold [10]

Myhrvold [10] investigated in his paper two hypotheses relevant for the studies of Werner and Griebeler [8] and of Grady et al. [7]:

“*H1*: *The metabolism of all members of a taxonomic group is determined by the regression parameters a and b for the group (from the allometric relationship G*_*max*_ = *aM*^*b*^*)*, *by comparison with a*, *b for groups with known metabolism*.” (with G_max_ maximum growth rate observed within a time unit throughout an individual’s ontogenetic growth, [44])
“*Statisticians refer to this problem as the “ecological fallacy”*: *the erroneous conclusion that one can infer individual properties from group-wide averages*.”
“*H2*: *The basal metabolic rate (BMR) is directly related to maximum growth rate G*_*max*_ *by an allometric equation BMR = αG*_*max*_^*β*^ *for constants α and β*.” As G_max_ scales with M, and BMR (only applicable to endotherms, in ectotherms the analogue is resting metabolic rate) scales with M, and both scaling exponents equal 0.75, Grady et al. [7] tested whether G_max_ and BMR statistically correlate (H2), thereby incorrectly assuming that statistical interference is transitive, which is another “ecological fallacy” [10].

Myhrvold [10] used G_max_ values of all extant ectothermic and of all extant endothermic vertebrate species in order to demonstrate that the thermal strategy of dinosaurs is not inferable from their maximum mass gain observed throughout ontogeny. He strongly disagrees in his paper with that we considered extant birds and reptiles (Sauropsida) as further reference models for dinosaurs. Contrary to the ectothermic and endothermic reference model, our Sauropsida model suggested that all studied dinosaurs had growth rates as seen in similar-sized (if necessary scaled-up) fast-growing ectotherms.

Myhrvold [10] additionally suggested in his paper that the mass-specific growth rate (kC = G_max_/M) should be preferred over the maximum growth rate G_max_ (termed maximum absolute growth rate in Werner and Griebeler [8]) when establishing scaling relationships on vertebrate growth rates. He therefore used a general model on ontogenetic growth ([55,56], a parametrisation of the standard models von Bertalanffy, Gompertz or logistic growth is possible in this model, G_max_ = kCM with k growth parameter and C constant, C differs between standard models). His method accounts for that the maximum growth rate (G_max_) used in all previous studies aiming at dinosaurian thermoregulation strategies is a function of (adult) body mass (M), because this introduces a correlation when regressing G_max_ against M. This correlation causes an inflation of the coefficient of determination R^2^ as well as a shrinking of confidence and prediction belts of regression lines. The latter conveys better fits of data to regression lines and could pretend no overlap between regressions on different groups. It is problematic when using confidence intervals of coefficients of regression lines to assess the separation of vertebrate groups (ectotherms and endotherms) and also when applying prediction belts of lines to infer whether a single dinosaurian G_max_ value is consistent with that of a similar-sized (if necessary scaled-up) animal from a given vertebrate group (or from ectotherms or endotherms). Myhrvold [10] shows that the use of his mass-specific growth rate (kC) leads to a stronger overlap in growth rates of vertebrate groups and in rates of endotherms and ectotherms, respectively, than the use of G_max_.

Throughout all his analyses regarding H1 and H2 as well as in his analyses on mass-specific growth rate (kC), Myhrvold [10] used ordinary least squares regression analyses (OLS). His only justification of preferring OLS over phylogenetically-informed methods was that OLS is “*sufficient to illustrate the phenomena discussed in the study*” [10].

## Our specific comments on Myhrvold [10]

Myhrvold criticized in his 2016 paper not only our study [8] but also that of Grady et al. [7]. In the following sections of this formal comment we mainly address those issues that Myhrvold [10] raised concerning our study [8], but also make here some remarks that are applicable to the Grady et al. [7] study.

### Ecological fallacy H1

We disagree with the critiques on our study given in Myhrvold [10] regarding H1. As described above, we did not infer any information on dinosaurian thermoregulation strategies from (vertebrate) group averages by comparing the dinosaurian regression line to those of different extant vertebrate groups. In fact, we already used Myhrvold’s [10] “parallelogram” method (explained above) to infer whether single dinosaurian G_max_ values are consistent with those of similar-sized (scaled-up) endotherms or ectotherms (fishes+reptiles vs. mammals+birds, see panel A of the first figure in our paper) or with those of similar-sized (if necessary scaled-up) ectothermic reptiles or endothermic birds (Sauropsida model, see panel B of first figure in our paper). This method is based on lower and upper maximum residuals and captures the maximum deviation of any studied species from the value expected under the group’s regression line from its mass. In the second figure of our paper [8] we show the variability seen in residuals (including the lower and upper maximum deviation from the average) for each vertebrate group as well as the overlap in G_max_ values of groups. Please note when referring to this figure that we could not reject a scaling exponent of 0.75 for any of the vertebrate groups studied. Thus, the intercept of each scaling regression sets the level at which residual variation is observed within the respective group and differences in group intercepts capture differences in the expected (from the group regression line) G_max_ values of similar-sized species from different vertebrate groups. We are aware of and agree with Myhrvold [10] on that our procedure assumes that all species pooled in a vertebrate group share similar characteristics in terms of physiology (e.g. thermoregulation, MR) and other biological characteristics or that at least variability in these is small compared the effect of body mass. We are also aware of that our approach using the lower and upper maximum deviation from the average strongly depends on sample sizes available for vertebrate groups. We expect, the larger the sample size, the larger the upper and lower deviation of maximum growth rates from the average. Anyhow, when comparing all extant endothermic to all extant ectothermic vertebrates our samples already demonstrated an overlap in G_max_ values of both groups and thus their sample sizes were unproblematic (second aim). Contrary, larger samples on endothermic and ectothermic Sauropsida could potentially demonstrate an overlap between reptilian and avian G_max_ values (third aim), which would reject our conclusion that dinosaurs had maximum growth rates as seen in similar-sized (if necessary scaled-up) fast-growing extant ectothermic reptiles. Our sample on reptiles (N = 35) was up to an order of magnitude smaller than that on precocial (N = 164) and altricial birds (N = 343).

### Ecological fallacy H2 and the Metabolic Theory of Ecology (MTE, [53])

We agree with Myhrvold [10] on that in any extant taxonomic group convincing evidence for a link between maximum growth rate and metabolic rate is still missing. We also agree with Myhrvold [10] on that the MTE is highly discussed, which provides a theoretical basis for a link between MR and G_max_ and also for the Grady et al. [7] study. Scaling exponents on the relation between MR and M differ between vertebrate groups (e.g. [57–59]), and even across reproduction strategies (e.g. [59]) and orders within a group (e.g. [60–62]). Nevertheless in our study from 2014, we found scaling exponents on maximum growth rate (0.75) which would support the MTE and we critically discussed this observation in our paper (Werner and Griebeler [8], see pages 6 and 7 therein). Authors making conclusions from G_max_ on MR argue that their relation is feasible as metabolism fuels growth (e.g. [15,63,64]). We found at least one study in the literature on endothermic birds providing experimental support at the level of individuals that both rates are indeed related. It showed that differences observed in G_max_ values of embryos of precocial and altricial birds that were incubated at an identical temperature correlated to differences in their MR values [65]. Nevertheless, besides the strikingly different metabolic situations seen in ectotherms and endotherms (see Introduction), growing birds and mammals, contrary to reptiles, pay extra metabolic costs of synthesizing new tissues beyond the metabolic costs that they have without growing (e.g. for foraging, digestion, production of new body substance [63]), and altricial birds and mammals show most of their growth when endothermy is not fully developed [66–69]. All these observations question the MTE as they indicate that differences in body temperature are not sufficient to fully explain differences in G_max_ and MRs of similar-sized growing ectotherms and endotherms [54].

Although, our analysis provides additional statistical support for the MTE (a scaling exponent of 0.75) and thus for a potential link between G_max_ and MR (but see contrary papers, e.g. [57–59] which question a single scaling exponent of 0.75 for MR in vertebrates and even within a vertebrate group), we have always carefully formulated our speculations on dinosaurian thermoregulation strategies and metabolic rates in our paper. We introduced our conclusions on dinosaurian MRs with the phrase “*Under the assumption that growth rate and metabolic rate are indeed linked*” [8] and noted in our discussion (amongst many other arguments against this link, see pages 6 and 7 in Werner and Griebeler [8]) that differences in G_max_ values of similar-sized extant vertebrate species are not consistent with those observed in their MR values as we would expect from the MTE. Additionally, Griebeler [54] had shown before that differences in G_max_ values of extant vertebrate groups do not only reflect differences in body temperatures of species. The (ectothermic) Arrhenius model used by the MTE to correct for differences in species’ body temperatures was not sufficient to explain differences in G_max_ values between similar-sized endothermic birds and mammals and could also not explain those between ectothermic reptiles and endothermic birds (mammals) [54]. Differences more likely reflect the strikingly different metabolic and growth situations seen in ectothermic and endothermic vertebrates (see Introduction). We thus wonder, why Myhrvold [10] states that our study presumes that a link between G_max_ and MR exists (H2).

Myhrvold [10] tried to reject the relationship between MR and G_max_ (H2) by reanalysing the original dataset from Grady et al. [7]. For extant vertebrate taxa, he found “*regressions between kC and M* (expected, scaling), *and between BMR and M* (expected, Kleiber’s law)*-but not between BMR and kC* (unexpected under the MTE)” [10], and he observed a clear separation in the scaling regressions on MR for endotherms and ectotherms, but not between regressions on kC (see sixth figure in Myhrvold [10]). We think his analysis does not clearly reject H2 for the following reasons. In their paper Grady et al. [7] chose only ectothermic species from tropical and subtropical climates or from laboratory settings between 24°C and 30°C and they adjusted the resting metabolic rates of species to 27 °C (= average of this temperature range). For mammals and birds these authors assumed that temperatures under which their BMR values (thermal neutral zone) and their G_max_ values were attained equalled the body temperatures of species [7]. The approach taken by Grady et al. [7] to assess thermoregulation strategies in dinosaurs is based on the MTE [53] and not on pure scaling arguments (H2, [10]). It assumes that the Arrhenius term in Eqs (1) and (2) cancels out when MR and G_max_ of each species are measured under equal temperatures. This is easily seen when reformulating scaling relationships on MR and G_max_ according to the MTE [53]. The joint effects of body mass M and temperature T_i_ (in K), on individual metabolic rate, MR, is described by

$$MR=i_{0}M^{0.75}\cdot e^{-E/k_{T_{i}}}$$ (1)

with i_0_ the normalization constant being independent of body mass and temperature, E activation energy, k Bolzmann’s constant, T_i_ temperature at which MR was measured for the individual, and $e^{-E/k_{T_{i}}}$ being the Arrhenius term. Analogously, the MTE formulates the joint effect of M and T_g_ on an individual’s G_max_ as

$$G_{\text{max}}=g_{0}M^{0.75}\cdot e^{-E/k_{Tg}}$$ (2)

Only if T_i_ equals T_g_ for an individual of a given body mass M any temperature effect on MR or G_max_ can be ignored and its

$$MR=\frac{i_{0}}{g_{0}}G_{\text{max}}\;\text{or}\;MR=\frac{i_{0}}{g_{0}}kCM,\;\text{respectively}.$$ (3)

For free-ranging ectothermic animals Grady et al. [7] used the long-term average annual air temperature seen in their habitats to adjust MRs in order to meet thermal conditions under which they grew. We think that this approach taken by Grady et al. [7] is flawed. As summarised in the introduction, the body temperature of ectotherms is the temperature to which the animal is currently exposed, and ectothermic animals are, at least to some extent, able to decouple their body temperature from the current air temperature seen in their habitat. Thus, body temperatures under which they grow must not equal ambient temperatures or even the average annual temperature of their habitat as assumed by Grady et al. [7] (see Introduction).

The clear separation in the scaling regressions on MR observed between endothermic and ectothermic species by Myhrvold [10] is a shear temperature effect in the raw data of Grady et al. [7] that he adopted for his analysis. Body temperatures of mammals and birds (e.g. range in mammals: 30.4–40.1°C, range in birds: 37.5–44.6°C; from Griebeler [54]) are considerably higher than the 27 °C to which these authors adjusted resting metabolic rates of ectothermic species. We thus expect the separation of regression lines on MR against M (sixth figure in Myhrvold [10]) simply from the temperature dependence of any chemical reaction.

A regression analysis on MR against G_max_ (kC) carried out consistent with the MTE in order to statistically test H2 requires that in each of the individuals both rates are measured under equal temperatures to ensure that in Eqs (1) and (2) T_i_ equals T_g_. We agree with Myhrvold [10] on his pure scaling argument that body masses at which MR and G_max_ values of individuals are measured must also be equal, so that in Eqs (1) and (2) the value of the term M^0.75^ is also equal and cancels out when equating MR and G_max_ (kC) as was done by Grady et al. [7]. All in all, a correct test whether MR and G_max_ are related requires an adjustment of both rates to the identical temperature for each species (for growth, temperature under which growth is monitored must be fixed to a specific value in the laboratory setting; for temperature, MR could be either measured under this specific temperature or if MR was measured under another temperature this rate could be adjusted to that under which growth was observed, for temperature adjustment methods of MR see e.g. White and Seymour [70]), and that both rates are measured for species at equal body masses. We are aware of that measurements on metabolic rates and growth rates in extant species that meet both assumptions (equal temperatures for growth and metabolic rate, equals body masses) are rare in the literature which obviously hampers any statistical test on whether MR and G_max_ are related. Anyhow, even when the Grady et al. [7] regression on MR and G_max_ would hold for extant species (Eq 3), its application to fossil taxa (e.g. dinosaurs) is problematic, as this requires accurate estimates of their body temperatures under which they attained G_max_ in order to estimate their MR. From a physiological perspective (see Introduction), the use of the average temperature in the Mesozoic as an estimate of dinosaurian body temperatures [7] is inappropriate to capture metabolic rates of any dinosaur (spawning several orders of magnitude in body size, covering different life styles, showing ectothermy including gigantothermy and endothermy in at least the bird lineage, …) that had lived in this era.

### Groups used as references for dinosaurs

**Fig 1:**
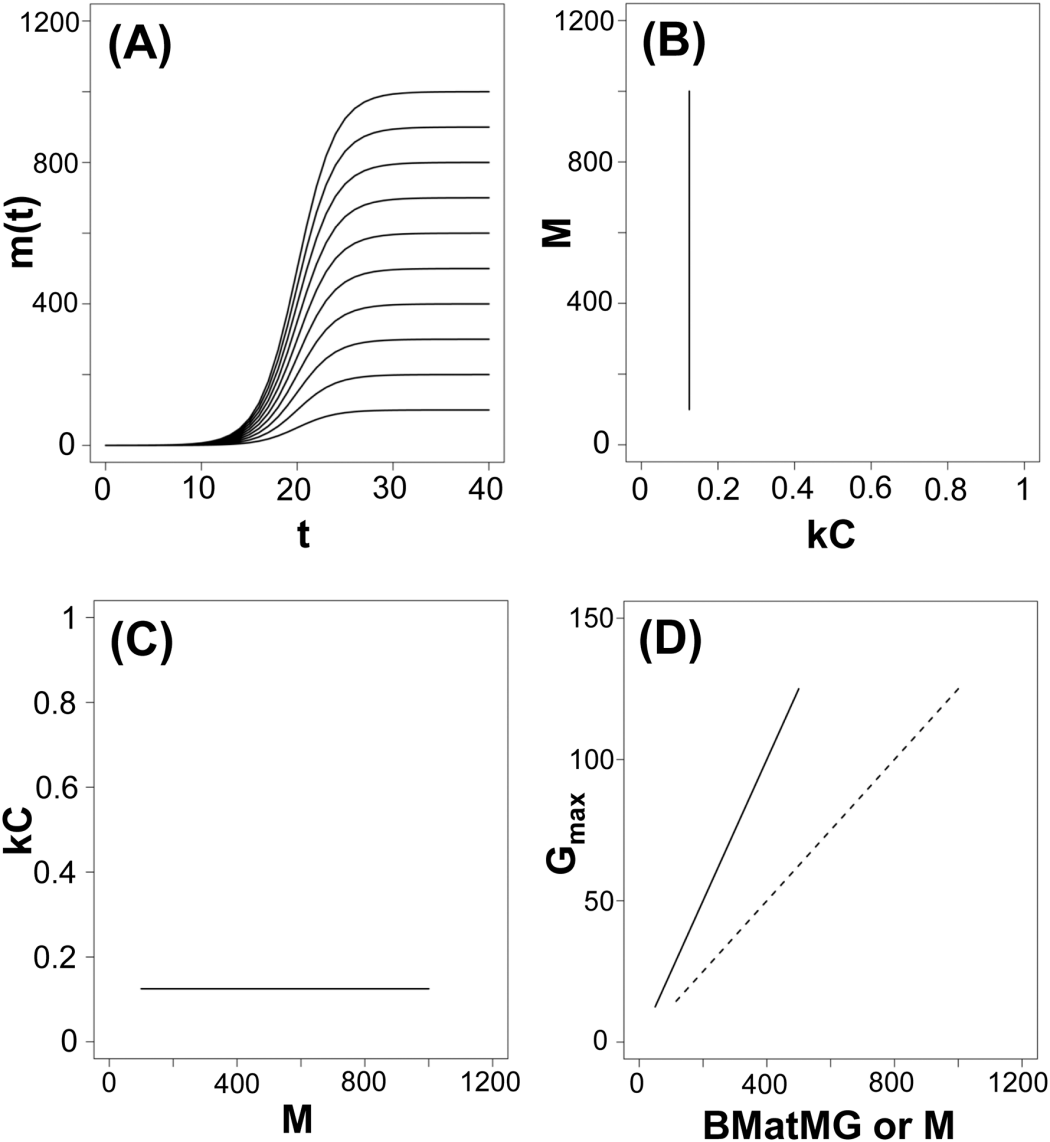
Comparison of the approaches of Case [44,45], Werner and Griebeler [8] and Myhrvold [10] to infer scaling regressions on maximum mass gain and asymptotic body mass for vertebrate taxa. We used the growth model as formulated in Myhrvold [10] (*m*(*t*) = *Mf*_log*istic*_(*s*) + *M*_*c*_ with $f_{\text{log}istic}\left(s\right)=\frac{1}{1+e^{-s}}$ and *s* = *k*(*t* − *t*_*c*_)) to establish logistic growth curves for 10 abstract species. Their asymptotic body masses (M) were 100, 200, 300, 400, 500, 600, 700, 800, 900, and 1000 mass units. The constant mass offset parameter M_c_ and the age offset parameter t_c_ in this model were set to 0. The growth parameter k with the dimension of inverse time was set to 0.5 for all species. Under this setting the maximum growth rate G_max_ of a species (= MkC, mass units per time unit) is M*0.5*0.25, as the dimensionless factor C is 0.25 for the logistic growth model. (A) Growth curves of species, m(t) evaluated for ages (t) 0 up to 40. (B) M is independent of kC. (C) Maximum mass gain versus body mass obtained under Myhrvold’s [10] kC-approach, no relation between M and kC. (D) Relationship when plotting G_max_ against BMatMG (body mass at which G_max_ is observed, Werner and Griebeler [8], 50% of M under a logistic growth model, solid line) or against M (dotted line, adult mass, approach of Case [44]), this is a unique relation between G_max_ and BMatMG (M), and it is consistent with the empirical observations that G_max_ values are higher in larger than in smaller species. Please note that for simplicity axes in (C) and (D) are not log-transformed.

Our study [8], that of Grady et al. [7] and previous paleontological studies (e.g. [11–15]) tried to assess dinosaurian thermoregulation strategies from maximum growth rates seen in different extant vertebrate groups. Myhrvold [10] disagrees with all these studies on considering different vertebrate groups as reference groups for dinosaurs and used in his study only the two reference groups all ectothermic and all endothermic vertebrates. His arguments were (1) that “*we cannot assume that it is legitimate to compare behavior-based groups* (precocial and altricial) *to lineage-based groups*” and (2) “*Either endothermy was an ancestral condition for the clade Dinosauria, or it evolved within the clade* (Fig 1). *In either case, one should expect a range of metabolic conditions and growth rates, at least some of which are likely to be endothermic*.” We suppose that his second argument expresses his aim to capture the large variability in metabolic conditions and growth rates seen in today’s ectothermic and endothermic vertebrates in order to assess dinosaurian thermoregulation strategies.

The use of extant and extinct species/taxonomic groups with morphological or other properties most similar to those seen in the fossils under study is a standard method applied in paleontology. From an evolutionary perspective, such reference models are based on the principle of convergence. Species filling similar ecological niches have similar morphological, physiological or other characteristics. This approach is problematic for the clade Dinosauria as a whole as they comprise a high variability in life styles (dinosaurs conquered aquatic habitats, terrestrial habitats, and the air), but feasible if a studied dinosaurian taxon has a life style that exists in extant (e.g. litter size in ichthyosaurs and in extant whales) or extinct vertebrate groups (e.g. litter size in ichthyosaurs and in plesiosaurs). A second approach often used to infer virtually any unpreserved trait of a fossil taxon with little or no modification over the evolutionary history is the Extant Phylogenetic Bracketing Approach [71]. Contrary to the first approach, it is based on homology and phylogenetic parsimony. It makes explicit reference to at least the first two extant outgroups of a fossil taxon under study in order to estimate a trait of interest. The second approach is not applicable to dinosaurs. Birds are living dinosaurs and thus they are no living outgroup. Endothermy arose in the dinosaurian lineage from an ectothermic ancestral state [17] and thus in the dinosaurian evolutionary history the thermoregulation strategy was strongly modified.

For the following phylogenetic and physiological reasons we chose modern reptiles and birds as reference groups for the 17 dinosaurs studied by us [8]. Extant birds and reptiles are phylogenetically closest to extinct dinosaurs, and birds are even living dinosaurs. Since endothermy evolved within the clade Dinosauria [17], extant reptiles are models for ectothermic dinosaurs (ancestral state), whereas birds are models for endothermic dinosaurs. With these two groups we aimed to capture the variability in maximum growth rates and thermoregulation strategies existing in the clade Dinosauria. Extant non-avian reptiles (crocodiles, turtles, squamates) and extant birds are Sauropsida, whereas extant mammals belong to the Synapsida. Sauropsida are a sister group of Synapsida with the latter giving rise to the Therapsida lineage, which in turn gave rise to extant mammals. Endothermy arose independently in the synapsid and therapsid lineage. This is still reflected in considerable differences in energy production and utilisation between extant birds and mammals [17]. The latter differences strongly question a use of extant mammals as reference group for extinct dinosaurs in order to assess their thermoregulation strategy. Moreover, dinosaurs share considerably more characteristics with birds and reptiles than they do with mammals or they do with any other extant vertebrate group (e.g. fish). This is because extinct dinosaurs and extant reptiles and birds have a long shared evolutionary history.

In our study we further distinguished between precocial and altricial birds. Contrary to precocial birds, in altricial birds the maximum growth rate is seen well before they fully achieve endothermy and this presumable resulted in higher growth rates in altricial than in precocial birds of similar size [40]. Our rationale was not to classify birds by behavioural differences as speculated in Myhrvold [10].

Myhrvold [10] further argues that the dinosaurs studied by us “*span both ornithischian and saurischian dinosaurs*” and that *Archaeopteryx* could be a bird, whereas all others are non-avian dinosaurs [10]. His arguments are only important in the light of H1 (see section “Ecological fallacy H1”), but we only used regression lines on vertebrate groups (including that on dinosaurs) to illustrate the variability in growth rates in each vertebrate group and their means (= intercepts). Anyhow, the G_max_ values of *Archaeopteryx* fit very well to our PGLS regression line on studied dinosaurs (see first figure in Werner and Griebeler [8]). This suggests that this putative bird species is no outlier with respect to its G_max_ to M relation when compared to the other dinosaurs studied by us. For our studied dinosaurs PGLS revealed a high λ value (0.933) which indicates a strong phylogenetic signal in the residual errors (the majority of dinosaurs analysed were sauropodomorphs and thus closely related compared to the others). The phylogenetic tree on dinosaurs used by us was constructed from the topology given in Lloyd et al. [72] and age ranges of taxa (branch lengths) were obtained from the Paleobiology Database [73]. We are aware of that this tree could not correctly reflect the evolutionary history of dinosaurs and could have affected our values of regression coefficients and thus the position of dinosaurian G_max_ values within extant vertebrates (see second figure in Werner and Griebeler [8]). However, PGLS is considered to be fairly robust with respect to errors in both phylogenetic topology and branch lengths [74].

### The kC-approach in Myhrvold [10]

Although, we appreciate very much the efforts in Myhrvold [10] to remove the correlation between G_max_ and M by using a mass-specific maximum growth rate (kC, analogous to the mass-specific metabolic rate), we think that this approach has important shortcomings.

**Fig 2:**
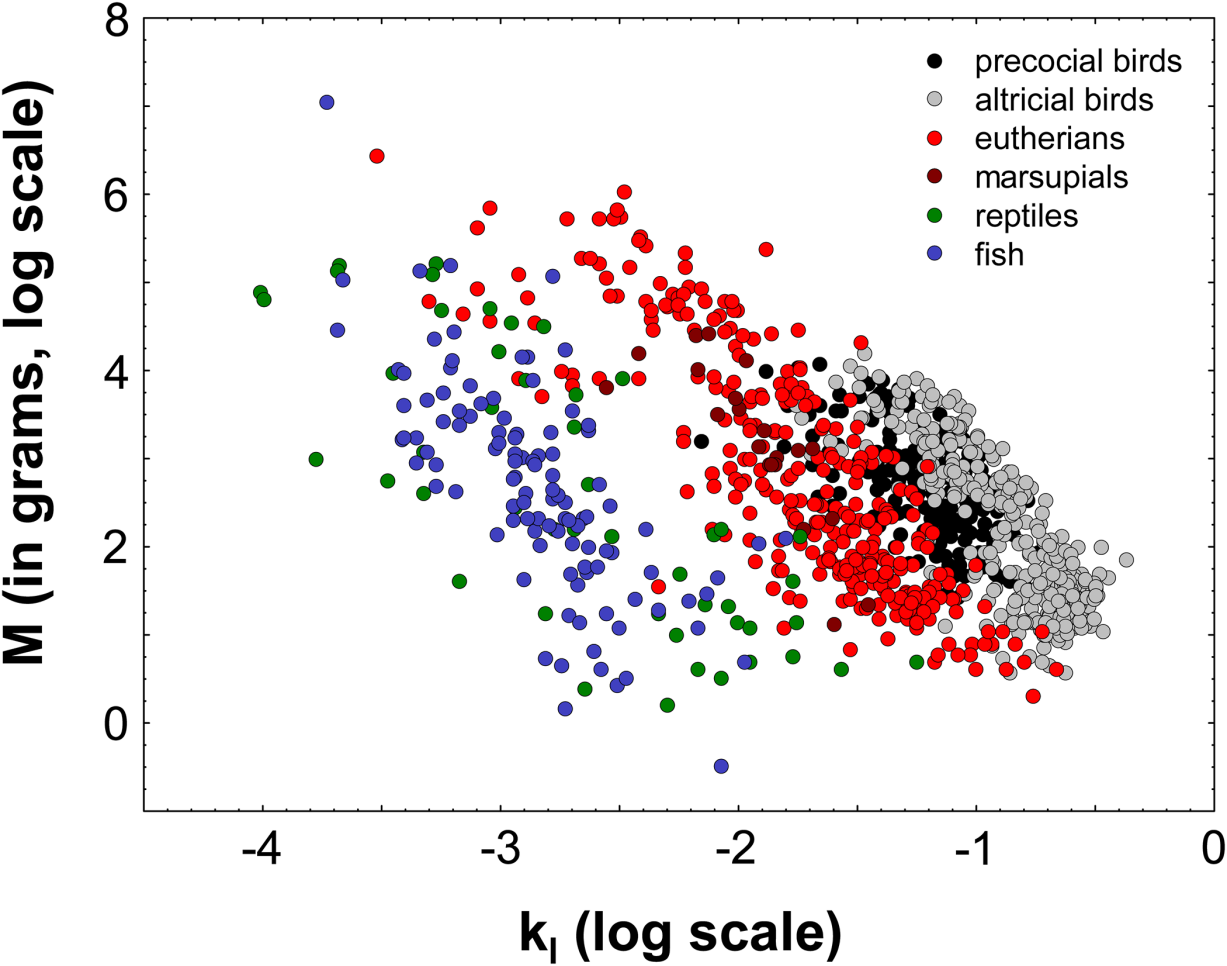
Growth curve constants and asymptotic body masses in different extant vertebrate taxa. We used growth data from Werner and Griebeler [8]. All k values of underlying growth models (von Bertalanffy, Gompertz or logistic growth model) were adjusted to the rate constant of the logistic equation (k_l_). Data on birds taken from Stark and Ricklefs [47], on mammals from Zullinger et al. [48], and on fish from Pauly [49]. Data on reptiles were compiled from literature for the study of Werner and Griebeler [8]. Species with an identical growth curve constant (k_l_) can strongly differ in their asymptotic body mass M, even if they belong to the same vertebrate group.

Dividing G_max_ by M (mass-specific maximum growth rate, increase in body mass per mass unit) completely reduces our information on an animal’s maximum body mass gain throughout its ontogeny to the percentage growth per unit time at the peak growth value observed (kC). We think that a good approach should work for any set of species taken from a group, whereas Myhrvold’s [10] approach does this not. Consider for this a set of species with an identical kC value that differ in their asymptotic masses (M, Fig 1A). Empirical studies have evidenced that species with identical kC values (or k values, if the identical growth model was applied to all species or if k values were adjusted to the rate constant of specific growth model) can indeed considerably differ in their asymptotic body masses [47–49] (Fig 2). Thus, when plotting pairs of M and kC for such species differing in M but not in kC, we obtain a line parallel to the y-axis (Fig 1B) and the scaling regression on kC for such species is a line parallel to the x-axis with the intercept kC (Fig 1C). The latter implies no effect of M on kC, and a scaling exponent of zero. Contrary, when plotting G_max_ against M or against BMatMG (body mass at which the maximum growth rate is observed, Werner and Griebeler [8], Fig 1d) for these species we have a unique relation between G_max_ and M (BMatMG). Contrary to kC versus M [10], for this set of species both scaling regressions reflect that maximum ontogenetic mass gain is higher in larger than in smaller species, and regressions now conform to empirical observations.

To establish our scaling relationships on G_max_ for different vertebrate groups, we preferred BMatMG, the body mass at which G_max_ is seen, over the asymptotic mass of the individual (M, [10,44]). The estimates on G_max_, M and BMatMG of species that we used in our study were extracted from growth models [8,47–49]. These models were established from growth data of several individuals of a species or even from single animals. Growth in ectotherms is highly plastic due to different environmental conditions experienced by animals (see Introduction, [75]), and also in endotherms (birds, [55,56]). Thus, even within a species, individuals of an identical adult body mass can considerably differ in their G_max_ values and vice versa. Thus, BMatMG is closer to the maximum growth ability of an individual than its asymptotic mass. G_max_ is attained at size BMatMG by the individual, and captures growth conditions at that time. Contrary, the asymptotic size M integrated growth and respective conditions over time (e.g. no growth under food shortage, under starvation animals can even shrink in mass). Furthermore, different standard growth models (von Bertalanffy, Gompertz or logistic growth model) were used to model ontogenetic growth of species. Standard growth models differ in the mass (BMatMG) at which G_max_ is observed even if birth masses and asymptotic masses do not differ between models. For fossils, BMatMG is better suited to establish scaling relationships on maximum mass gain than asymptotic size (M). When we have a good mass estimate for the fossil under study, its BMatMG is always documented in its growth record together with G_max_, irrespective of whether a good estimate on its asymptotic mass is available, too (i.e. the animal is fully-grown or a growth model was successfully applied to the specimen in order to estimate M). In taxa forming ontogenetic growth marks, growth records are often incomplete with respect to the three phases covered by sigmoidal growth [16,76–78], which hampers the establishment of reliable growth models for fossils. For example, the individual died before it reached the asymptotic phase of growth and/or its bone shows remodelling in the inner part leaving only few peripheral growth marks behind (e.g. [16]).

### Ordinary vs. phylogenetically-informed least squares regression analysis

Throughout all his analyses Myhrvold [10] used ordinary least squares regression analyses (OLS). Contrary, the study of Werner and Griebeler [8], used both phylogenetically-informed and OLS analysis (the latter in order to figure out whether Case’s regressions [44] were reproduced with our larger dataset on vertebrates, first aim). We finally found out that both regression methods revealed consistent results on scaling relationships on maximum ontogenetic mass gain (G_max_ against BMatMG) and thermoregulation strategies of studied dinosaurs (see the Supporting Information of Werner and Griebeler [8]).

Phylogenetically-informed regression analysis aims at the statistical dependence of species traits that originates from a shared evolutionary history of species analysed [74,79,80]. The shared evolutionary history implies a covariation in traits across species. Closely related species are expected to produce more similar residuals from the least squares regression line than less closely related. Thus, residuals are not statistically independent and OLS assumptions are violated. Under the phylogenetic generalised least squares regression analysis (PGLS, [50–52]) applied by us the phylogeny is used to calculate the expected covariance structure in the data. Using phylogenetically dependent data in OLS analysis could reveal wrong estimates for regression coefficients, causes Type 1 error inflation, could lead to wrong standard errors of estimated coefficients, and to wrong confidence and prediction belts of regressions [79,80]. We thus wonder, why Myhrvold [10] focused on the correlation between G_max_ and M, but ignored this further important and well-known source of error [79,80]. For our scaling relationships on G_max_, the λ values estimated under PGLS ([50–52], 0 ≤ λ ≤ 1) were considerable higher than zero (see Table 1 in Werner and Griebeler [8]). They thus indicated that PGLS removed a strong phylogenetic signal in the residual errors for the vertebrate groups studied by us. Except for marsupials (λ = -0.305, N = 21, the small sample presumably hampered a correct co-estimation of the evolutionary model parametrized by λ), λ values ranged from 0.543 to 0.945 in extant vertebrate groups and λ was 0.933 for dinosaurs. Estimated λ values being larger than zero thus clearly justified our use of phylogenetically-informed regression analyses. That our OLS analysis and PGLS analysis revealed similar results when regressing G_max_ against BMatMG does not imply that this is also true for kC versus M as assumed in Myhrvold [10]. PGLS takes into consideration the covariance structure in residuals which in this case originates from regressing kC against M. Likewise, he cannot assume that controlling for the shared evolutionary history of species is redundant for MR against kC, when OLS and PGLS had revealed similar results when regressing MR against G_max_ [7].

## Conclusions

None of the critiques raised in Myhrvold [10] and referred to as H1 and H2 in his paper is justified for ours [8]. We did neither infer the thermoregulation strategies of our studied 17 dinosaurs from group-wide averages (H1) nor was our study based on that G_max_ and MR are related (H2). We agree with Myhrvold [10] on the build-in correlation between G_max_ and M, but showed here that the use of his mass-specific maximum growth rate (kC) instead of the maximum growth rate (G_max_ = MkC) introduced by Case [44,45] and used in many subsequent studies, comes at the shortcoming that the empirically observed scaling relationship between G_max_ and M is not modelled for any set of species taken from a group. In this formal comment we also explained our use of the Sauropsida model for dinosaurs, our use of BMatMG in scaling relationships on maximum mass gain and our use of phylogenetically-informed regression analysis. We also refuted a number of supporting arguments given in Myhrvold [10].
